# NRXN1 Deletion in Two Twins’ Genotype and Phenotype: A Clinical Case and Literature Review

**DOI:** 10.3390/children9050698

**Published:** 2022-05-10

**Authors:** Monica Sciacca, Lidia Marino, Giovanna Vitaliti, Raffaele Falsaperla, Silvia Marino

**Affiliations:** 1 Department of Clinical and Experimental Medicine, Section of Pediatrics and Child Neuropsychiatry, University of Catania, 95100 Catania, Italy; doc.monica.sciacca.ms@gmail.com (M.S.); lidia.m@hotmail.it (L.M.); 2 Section of Pediatrics, Department of Medical Sciences, Sant’Anna University Hospital, University of Ferrara, 44121 Ferrara, Italy; 3 Neonatal Intensive Care Unit, AOU “Policlinico”, PO “San Marco”, University of Catania, 95100 Catania, Italy; raffaelefalsaperla@gmail.com; 4 Unit of Pediatrics and Pediatric Emergency, AOU “Policlinico”, PO “San Marco”, University of Catania, 95100 Catania, Italy; silvia_marino86@hotmail.it

**Keywords:** children, neurexin, NRXN1, neurodevelopmental delay, genetic analysis

## Abstract

In the literature, deletions in the 2p16.3 region of the neurexin gene (NRXN1) are associated with cognitive impairment, and other neuropsychiatric disorders, such as schizophrenia, autism, and Pitt–Hopkins-like syndrome 2. In this paper, we present twins with deletion 2p16.3 of the NRXN1 gene using a comparative genomic hybridization array. The two children had a dual diagnosis consisting of mild cognitive impairment and neurodevelopmental delay. Furthermore, they showed a dysmorphic phenotype characterized by facio-cranial disproportion, turricephalus, macrocrania, macrosomia, strabismus, and abnormal conformation of both auricles with low implantation. The genetic analysis of the family members showed the presence, in the father’s genetic test, of a microdeletion of the short arm of chromosome 2, in the 2p16.3 region. Our case report can expand the knowledge on the genotype–phenotype association in carriers of 2p16.3 deletion and for genetic counseling that could help in the prevention and eventual treatment of this genetic condition. Newborn carriers should undergo neurobehavioral follow-ups for timely detection of warning signs.

## 1. Introduction

Neurexins are pre-synaptic cell adhesion molecules that are fundamental for promoting and maintaining synaptic connections between various brain structures [1].

The neurexin 1 gene, which has been mapped on chromosome 2, encodes for neurexin, which is a cell adhesion molecule and also acts as a receptor in the vertebrate nervous system. The binding of neurexin at a presynaptic level with neuroligin located at the post-synaptic membrane plays a key role in the development of a correct synaptogenesis process, also important for promoting the cellular adhesion mechanism [2]. The binding of the neurexin with the neuroligin ensures the correct alignment and positioning of the synaptic button and this also allows the right interlocking between the neurotransmitter released in the synaptic wall with the post-synaptic receptor [2].

According to the literature, deletions of chromosome 2 at the 2p16.3 region—and therefore, the Neurexin gene—have been associated with the presence of intellectual disability and other neuropsychiatric disorders, such as autism spectrum disorders (ASD), attention deficit hyperactivity disorder (ADHD), seizures, schizophrenia and bipolar disorder, and Pitt–Hopkins-like syndrome 2 [3,4,5,6,7,8,9,10,11,12,13,14,15,16,17].

Pitt–Hopkins-like syndrome 2, described in literature as NRXN1 mono-bi-allelic deficiency, constitutes one of the most representative phenotypes associated with NRXN1 defects. In this condition, compound heterozygous inherited NRXN1 deletions/mutations produce a mild to severe recessive phenotype, including moderate or severe intellectual disability, speech impairment, motor hypotonia and stereotypy, impaired sleep–wake cycle, and constipation [17].

## 2. Report

We present two small twin patients with intragenic 2p16.3 deletion of the NRXN1 gene using a comparative genomic hybridization array (CGH array).

The two babies were conceived through in vitro fertilization (IVF) at the first induction cycle and after 5 years of unprotected intercourse to achieve a natural pregnancy. The mother had severe obesity with a BMI of 40 and no other noteworthy pathology. No abnormalities of the ovarian cycle and ovarian reserve were reported. The father was affected by oligospermia in the absence of further organic pathologies, for which the doctors decided to undertake medically assisted procreation therapies. The mother denies the use of any drug during pregnancy other than multivitamin supplements based on folic acid, chiro inositol, and probiotics. No states of hypertension, gestational diabetes, and preeclampsia have been reported during pregnancy. At about 25 weeks, the woman underwent hospitalization for early onset of uterine contractions. The two small patients were born at 26 + 5 weeks of gestation due to a placental abruption that required an emergency cesarean section.

### 2.1. Case 1

The first baby, born at 26 weeks and 5 days of gestation, presented healthy clinical conditions at birth. The birth weight was 1135 g, length 35 cm, head circumference 24 cm. A single umbilical artery was found. A few minutes after birth, he was resuscitated due to cardio-respiratory depression, for which he was placed in nCPAP FiO_2_ 30% and was transferred to NICU, where he underwent cardio-respiratory monitoring. Following this, he was intubated and ventilated in SIMV-ET mode. Surfactant was also administered. The baby was extubated after about 5 days but, due to clinical worsening, he again underwent to orotracheal intubation. He then moved on to non-invasive ventilation and finally to high-flow oxygen therapy until the 20th day of hospitalization. The child also presented severe hypotension for which it was necessary to administer vasoactive amines for about a week.

During hospitalization, he performed phototherapy for preterm jaundice, erythropoietin therapy, and blood transfusions for anemia. At the cerebral ultrasound scan, he presented left side fourth degree IVH cerebral hemorrhage in ex vacuo poroencephalic hydrocephalus. Moreover, during hospitalization, he presented epileptic events characterized by general tonic–clonic movements that required a treatment with Luminale. The video-EEG examinations performed at one month showed a low base voltage and sharp theta on the left center-temporal regions, but these anomalies normalized at follow-up EEGs.

For the persistence of hypotonia and the presence of seizures, the child underwent brain MRI at about one month of age and presented outcomes of hemorrhage with ex vacuo dilation of the left lateral ventricle from a large left fronto–parieto–occipital malacic area. Triventricular hydrocephalic dilation and thinned corpus callosum were also observed. Echocardiographic evaluations, renal ultrasound, and chest–abdomen graphic X-ray investigations did not show significant alterations, considering the prematurity and in relation to the general clinical conditions.

At about two-and-a-half months of life, a physiatry consultation was performed which showed his head slightly oriented to the left and that left plagiocephaly was present. During sleep, the child had frequent desaturations without the need for oxygen administration. He presented weakness of the right upper limb and mild hypotonia in the right side as well. The neurological evaluation performed at about 4 months of life showed: turricephalic aspect of the head, visual attachment was present but inconstant, and axial hypotonus slight hypotonus of the right side which was more marked at the level of the upper limb. Moreover, the baby performed a further cerebral ultrasound examination which showed persistence of the porencephaly area of the left hemisphere as a result of previous IVH grade IV and mild ventriculomegaly of the right lateral ventricle. At about one year of age, the baby underwent further neuropediatric evaluation which showed the persistence of plagiocephaly, dysmorphic face, and low-set ears with associated abnormal conformation. The assessment of muscle tone highlighted some difficulties in keeping his head aligned and, in general, the presence of axial hypotonia. Orthoptic evaluation revealed epicanthus and esotropia, and ophthalmological evaluation of the fundus oculi showed the presence of a pale papilla in the temporal sector.

The first little twin showed muscle weakness, hypotonia, and reduced spontaneous activity from birth. Psychomotor development was delayed: postural control of the head and trunk occurred at 8 and 14 months respectively; there was also a motor coordination deficit. Babbling started at 10 months without ever progressing into expressive language. The patient was also wise in non-verbal communication. There were frequent stereotyped movements of the upper extremities for visual self-stimulation. At the call of his name, the patient turned around with an improvement in his visual development over time. His sleep–wake cycle was disturbed by frequent nocturnal awakenings.

**Figure 1:**
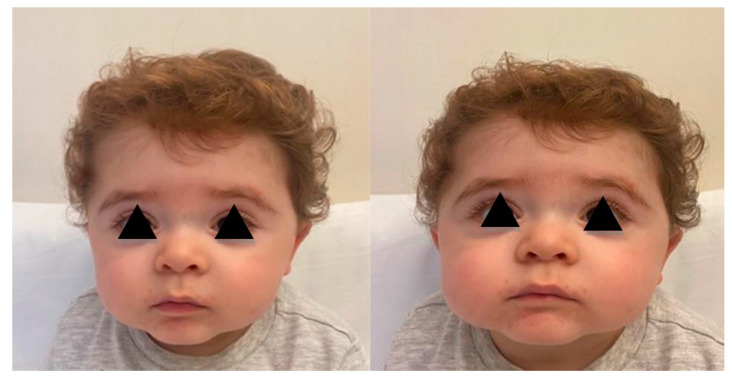
Case 1: Cranial and facial dismorphysm.

At the current evaluation, the child presented facial dysmorphism including depressed nasal spine, craniofacial disproportion, macrocrania, macrosomia, mild turricephalus, epicanthus, strabismus, frontal protuberance with high anterior hairline, ‘cupped’ auricles with low implantation, slightly prominent glabella, and infraorbital folds (Figure 1).

No dysmorphisms were present in other corporeal areas, such as the trunk and limbs.

### 2.2. Case 2

The second baby, the twin brother, born at 26 weeks and 5 days, at birth presented fair conditions as well. The birth weight was 1010 g, length 33 cm, head circumference 23 cm. A few minutes after birth he presented cardio-respiratory depression requiring positive pressure ventilation and 40% FiO_2_. After stabilization of the vital signs, n-CPAP was positioned with FiO_2_ at 30% and the baby was transferred to NICU where he underwent cardio-respiratory monitoring, endotracheal intubation and ventilated in SIMV-ET mode. Surfactant was also administered. The baby was extubated after about 24 h; however, due to frequent crises of desaturation and bradycardia, he was repeatedly reintubated in the following days; then he was placed in n-CPAP, high flow oxygen therapy, and finally passed into spontaneous breathing. During the hospitalization the baby presented hypotensive events, hyperbilirubinemia for which the necessary treatments were carried out. Alterations in coagulation factors and anemia were treated by transfusions with plasma and concentrated red blood cells.

At physical examination, the second little twin presented muscle weakness, hypotonia, and reduced spontaneous activity since his birth as well. Furthermore, in this case, there was a delay in psychomotor development. Postural control of the head and trunk occurred at 9 and 14 months, respectively, with impaired motor coordination. At 9 months, the lallation began without evolution in expressive language.

**Figure 2:**
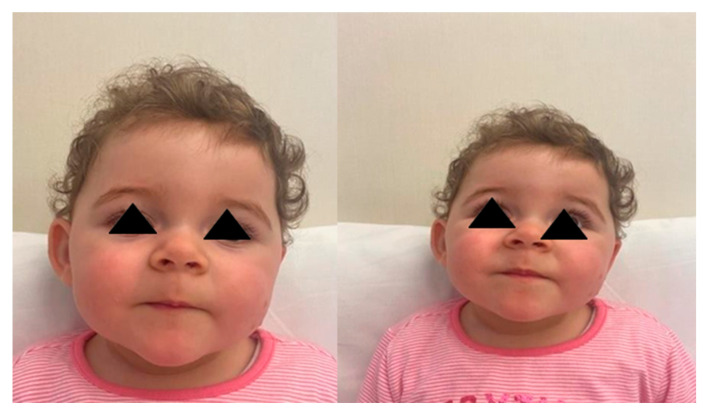
Case 2: Cranial and facial dismorphysm.

A cerebral ultrasound scan performed after birth showed a third-degree intraventricular hemorrhage IVH III which was subsequently reabsorbed. For the presence of this important hypotonia, brain MRI was performed at about two months of life and showed no noteworthy alteration except a small picture of hem ventricle affecting the posterior horn of the right lateral ventricle in a patient with previous triventricular hemorrhage. ACGH array genomic investigation was also performed and it showed the same alterations highlighted in the little brother and described above. The child neurodevelopmental outcome revealed an absent non-verbal communication, including pointing. Stereotypical hand movements for visual self-stimulation were frequent. When called by name, the patient turned around and his visual involvement has improved over time. Finally, his sleep–wake cycle was disturbed by nocturnal awakenings. At the current evaluation, the second child also presented faciocranial disproportion, macrocrania, macrosomia, retrognathia, and tended to tilt the head to the left side due to persistence of axial muscle hypotonia (Figure 2). No dysmorphisms are present in trunk and limbs. Feet were normal.

### 2.3. Psychomotor Development of the Two Babies

We performed an appropriate follow-up of the psychomotor development of our children, by using the Hammersmith Neonatal Neurological Examination (HNNE), the Hammersmith Infant Neurological Examination (HINE), and the Griffiths scale. The two babies underwent a clinical and diagnostic evaluation at 3 months (T1), 6 months (T2), 9 months (T3), and 12 months (T4) to assess psychomotor development, and the Griffiths scale score system15 (12 mo) to assess development quotient. The HNNE performed at T0 showed suboptimal values (Table 1). At 3 months of age, we recorded a suboptimal global score in posture, movements, tone and reflexes, and cranial nerves. At 6, 9, and 12 months of age, the babies had suboptimal values on the HINE as well. At 12 months, they had low-average Griffiths scale scores.

### 2.4. Genetic Analyses of the Two Babies and Their Parents

Genomic analysis using CGH-array revealed an interstitial microdeletion of the short arm of a chromosome 2 in the 2p16.3 region, extending approximately 157 kb. The deleted genomic region is intragenic to the NRXN1. Genomic analysis using CGH array, performed on female sexual complement DNA of the mother, did not show microdeletions and/or microduplications in the analyzed sample. Genomic analysis by CGH array, performed on male sexual completion DNA of the father, revealed an interstitial microdeletion of the short arm of chromosome 2 in the 2p16.3 region, extending approximately 157 kb. The deleted genomic region is compatible with the rearrangement found in the off-spring by the same investigation. The father of the two babies had the same deletion, but in a mild form and without dysmorphism. The most serious clinical phenotype presented by the children could be explained by the epigenetic alterations induced by prematurity and by all the consequences that this has brought in the clinic of the two children and in their subsequent neurodevelopmental stages.

## 3. Discussion

In the literature, other patients with cognitive, clinical, neurodevelopmental abnormalities, and dysmorphic phenotype, who have genomic alterations producing heterozygous NRXN1 loss of function have been described. In the study of Ching et al. the overall frequency of NRXN1 exonic deletions has been reported as 0.019% in control populations [3]. It has also been shown that NRXN1 microdeletions can be inherited even by apparently healthy parents [4]. For this reason, a role of the multifactorial, polygenic, and epigenetic effect in the expression and penetrance of NRXN1 has been hypothesized; in fact, the etiopathogenetic mechanism of the “second hit” has been proposed for other disorders linked to the presence of copy number variants (CNV) [5,6,7]. However, only limited clinical information is available; therefore, it is not possible to make a phenotypic comparison with our case (Table 2).

Zahir et al. described one case with mild mental retardation, autistic features, multiple vertebral malformations, and an unusual facial appearance who carries a de novo submicroscopic deletion of chromosome 2p16.3 [8]. Vinas-Jornet et al. reported three cases of unrelated patients, two adults and one child, in whom they identified an intragenic 2p16.3 deletion within the NRXN1 gene. The three patients presented dual diagnosis that consisted of mild intellectual disability, autism, and bipolar disorder. Furthermore, they all shared a dysmorphic phenotype characterized by a long face, deep-set eyes, and prominent pre-maxilla [9]. Bermudez-Wagner et al. presented a patient with dysmorphic features, hypostature with microcephaly, hypotonia, and a persistent patent ductus arteriosus with global developmental delay associated an exonal deletions of NRXN1 [10]. Alfieri et al. described the cognitive and behavioral profiles of five children with a heterozygous NRXN1 deletion, studied through a systematic assessment of cognitive and developmental levels, of the adaptive profile and of the presence of behavioral symptoms and/or autistic characteristics. In addition, four out of five transmitting parents were assessed by means of cognitive, psycho-pathological, and parental stress tests. They documented a below-average cognitive level in all children, and observed in four of them defective adaptive levels. Three out of five children were diagnosed with ASD associated with intellectual disability, and global developmental delay with severe communication impairment [11]. These findings support the idea that a-1-neurexin is necessary for a normal neurological development and suggests that this process could be related to the extension of NRXN1a alteration. The dysfunction of the neurexins results in the inability of the synapses to transmit signals between neurons, however without a complete collapse. This detail justifies the fact that individuals with the same deletion or mutation, even if belonging to the same family, show different phenotypes [15].

The clinical presentation of the two little babies includes neurodevelopmental abnormalities and dysmorphic profile. A moderate to severe intellectual cognitive impairment, the alteration of expressive language, muscle hypotonia, and abnormal sleep/wake cycle have been described in our report as well. The pathological psychomotor and cognitive-behavioral development found in the little twins could fall within the characteristics of the Pitt–Hopkins-like syndrome 2, but unfortunately this syndrome genotype—as described so far in the literature—does not match with the interstitial microdeletion of the short arm of chromosome 2 in the 2p16.3 region found in our case report. In particular, the mutations related to Pitt–Hopkins-like syndrome 2 and described so far in literature were mono or bi-allelic exonic NRXN1 deletions [17].

Nevertheless, in our case report we confirm—according to the literature—the wide phenotypical variability observed in individuals with intragenic deletions affecting NRXN1, documenting cognitive impairment ranging from mild to severe global developmental disorder, neurodevelopmental delay, and dysmorphic profiles. Unfortunately, we can state, referring to both the literature and our case report, that children with intragenic heterozygous NRXN1 deletions do not show specific clinical patterns and the variety of clinical phenotypes linked to this mutation prevents us from associating an appropriate predictive power to the mutation in future clinical developments.

## 4. Conclusions

The cognitive and dysmorphic phenotype found in carriers of 2p16.3 deletion, and described in our case report, suggests that 2p16.3 deletions could have variable expressiveness and incomplete penetrance which, unfortunately, even after prenatal detection of the deletion it is possible to have reliable predictions on clinical outcomes [16].

Furthermore, prognosis may be influenced by other independent genetic risk factors that should be sought [17].

At the same time, it is important to describe and implement genetic and clinical research on the subject in order to more selectively associate the presence of this mutation with the clinical characteristics of the carriers. Furthermore, our case report may expand as-yet limited knowledge on genotype–phenotype association in carriers of 2p16.3 deletion. Therefore, together with the literature, it is possible to provide information on the possible phenotype and direct genetic counseling to approach the patient as early as possible and in the best possible way. Newborn carriers should undergo neurobehavioral follow-ups for early detection of warning signs and respond quickly.

## Figures and Tables

**Table 1:** Hammersmith Infant Neurological Examinations (HINE) of case reports.

	HINE (Global Score)	Cranial Nerves	Posture	Movements	Muscle Tone	Reflexes and Reactions
**Case 1**	67	14	16	5	18	14
**Case 2**	68	13	18	5	17	15

**Table 2:** Details of other reported cases with genomic aberrations expected to cause NRXN1 loss of function.

Case	Source	Genomic Features	Method	Reported Phenotype and Clinical Evaluation
Case 1	F R Zahir et al. [8]	Submicroscopic deletion of chromosome 2p16.3, 320 kb in size, and includes only the part of the NRXN1 gene that codes for the neurexin1a promoter and initial coding exons	Array genomic hybridisation (AGH)	Mild mental retardation, autistic features, multiple vertebral malformations, and an unusual facial appearance
Case 2	Marina Viñas-Jornet et al. [9]	Intragenic 2p16.3 deletion within the NRXN1	CGH array	Bipolar disorder. IQ of 65. Poor behavioral control, difficulty in acquiring new information, both verbal and visual. Facial dysmorphism: long face, deep-set eyes, hypotelorism, low set ears, prominent premaxilla, a high, narrow palate, and tooth malposition. Dorsal kyphosis and long hands with slender, flexible fingers.
Case 3	Marina Viñas-Jornet et al. [9]	Intragenic 2p16.3 deletion within the NRXN1	CGH array	Facial dysmorphism, long face, deep-set eyes, hypotelorism, low set ears, prominent premaxilla, and high palate; dorsal kyphosis and finger rigidity. Behavior abnormalities included explosive temper tantrums, violence, and property destruction with a diagnosis of verbal and physically aggressive destructive behavior. IQ of 65
Case 4	Marina Viñas-Jornet et al. [9]	Intragenic 2p16.3 deletion within the NRXN1	CGH array	Dysmorphism with a mildly long face, deep-set eyes, prominent premaxilla, and long philtrum. Autistic traits, with hyperactivity and challenging behavior as his most salient psychopathological features. IQ of 53 and a neuropsychological profile characterized by language impairment (both expression and comprehension), poor working memory, and attention.
Case 5	Karla Bermudez- Wagner et al. [10]	2p16.3 microdeletion with partial deletion of the neurexin-1 gene	CGH array	Morgagni diaphragmatic hernia developmental delays hypotonia, short stature, ptosis, wide mouth, brachydactyly nail hypoplasia
Case 6	Paolo Alfieri et al. [11]	103.5 Kb deletion at 2p16.3 com- prising one NRXN1 exon	CHG array	Cognitive/developmental delay ASD
Case 7	Paolo Alfieri et al. [11]	Deletion spanning 324.3 Kb including four NRXN1 exons	CGH array	Cognitive/developmental delay ASD
Case 8	Paolo Alfieri et al. [11]	Microdeletion spanning 72 Kb and involving four NRXN1 exons	CGH array	Cognitive/developmental delay
Case 9	Paolo Alfieri et al. [11]	1.5 Mb deletion, which included three NRXN1 exons	CGH array	Emotional and behavioral problems cognitive/developmental delay ASD
Case 10	Paolo Alfieri et al. [11]	Deletion spanning 144 kb including one NRXN1 intron	CGH array	Cognitive/developmental delay ASD

## Data Availability

Data are available on request to the corresponding author.
